# Crowdsourcing Our National Gut

**DOI:** 10.1128/mSystems.00060-18

**Published:** 2018-05-15

**Authors:** Laura E. Grieneisen, Ran Blekhman

**Affiliations:** a Department of Genetics, Cell Biology, and Development, University of Minnesota, Minneapolis, Minnesota, USA; b Department of Ecology, Evolution, and Behavior, University of Minnesota, Minneapolis, Minnesota, USA

**Keywords:** American Gut Project, citizen science, gut microbiome, metabolome

## Abstract

The microbes of the human intestinal tract play a profound role in our health. The complex interactions between our gut microbial communities and the external environment, and the resulting functional consequences, can be difficult to disentangle. To address this problem, McDonald et al. (mSystems 3:e00031-18, 2018, https://doi.org/10.1128/mSystems.00031-18) present the first set of results from the American Gut Project, a citizen science-based data set currently comprised of over 10,000 gut microbiome samples and associated life history data. By combining this extensive data set with other published studies, the authors uncover novel relationships between gut microbiome structure and function. For example, they found that dietary plant diversity and recent antibiotic use predict both microbial and metabolomic diversity. McDonald et al. also demonstrate that there is high diversity across human gut microbiomes, even compared to the diversity of environmental microbiomes. The results from this study illuminate the potential of the citizen science approach to further our knowledge of host-associated microbial communities.

## Abstract (precis)

The microbes of the human intestinal tract play a profound role in our health. The complex interactions between our gut microbial communities and the external environment, and the resulting functional consequences, can be difficult to disentangle.

## COMMENTARY

Variation in gut microbiome composition, defined as the number and types of microbes living in the human digestive tract, is increasingly linked to human health and physical functioning. Such effects include changes in metabolism, hormone levels, obesity, and autoimmune diseases (1–4). Understanding how a person’s behaviors, diet, and environment shape the composition of the gut microbiota and how these interactions in turn are linked to downstream physiological consequences is a central goal in human health research (5, 6). However, such relationships are difficult to tease apart, as many studies are limited by small sample sizes, a lack of diversity in geographic location and other environmental characteristics, and limited information on the life history traits of study subjects. In their article, McDonald et al. (7) address these gaps by combining gut microbiome samples submitted by over 10,000 citizen scientist contributors with published data sets and environmental samples from the Earth Microbiome Project. They survey gut microbial differences between human populations and between human and environmental microbiome samples and test how dietary diversity and antibiotic use predict microbial and metabolomic diversity. By making novel connections across data sets and leveraging citizen science data, McDonald et al. implement a research model likely to be key in future human microbiome analyses.

An increasing number of projects are using citizen science approaches both to increase sample size and diversity and to provide members of the public with an opportunity to learn about the natural world and participate in the scientific endeavor. Successful citizen science projects often have a conservation angle; for example, members of the public have contributed to over 200 articles as they tracked avian migration patterns over the last century (http://www.audubon.org/christmas-bird-count-bibliography), documented the appearance and spread of invasive species (8), and quantified predation of native wildlife by housecats (9; http://www.kittycams.uga.edu/). Other studies rely on crowdsourcing to complete computationally intensive tasks, such as protein folding (10; https://fold.it/portal/info/about) and searching satellite imagery for unexcavated archaeological sites (https://www.globalxplorer.org/about). More recently, citizen science projects have begun to focus on microbial communities and health, allowing participants to learn about their local microbiota (http://robdunnlab.com/projects/wild-life-of-our-homes/) and to get health-related feedback on their microbial communities (11; uBiome). The American Gut Project is a large-scale citizen science project that provides participants with a personalized profile of their gut microbial communities while also providing researchers with thousands of samples and associated metadata to run powerful analyses.

The results from the current study highlight a broad survey of the types of microbiome analyses made possible by combining a citizen science data set with other published studies. Some of the main outcomes support results from previous studies; for example, the gut microbiome of individuals living in the United States and the United Kingdom differed from each other, and both differed from those of hunter-gatherers and other populations living traditional lifestyles. An individual’s microbiome remained relatively stable over time, and geographic distance between populations did not predict microbial distance. Other results provide novel insights into linking microbiome composition with function. Metabolomic analyses yielded two amides that correlate with thyroid disease and revealed that recent antibiotic use predicted high molecular diversity but low microbial diversity. Although dietary categories like “omnivore” or “vegan” did not predict microbiome clustering, using a more fine-scale approach of dietary plant diversity showed differences in both microbial and molecular diversity. Finally, the large data set suggests the importance of large sample sizes in microbiome studies; there was a reduction in the discovery of new microbes at ~3,000 samples, and, perhaps surprisingly, variation between gut microbiomes in the American Gut Project was greater than the variation between diverse environmental microbiomes from the Earth Microbiome Project data set.

Undergoing such a broad analysis is not without its challenges, some of which are technical and some of which are inherent to citizen science projects. First, there is an issue with data quality. Unlike other microbiome studies, which recommend freezing samples or preserving them in liquid (12,13), the current study used stool samples collected with dry swabs. Although this is a more convenient collection method that can increase participation in the project, not using a preservative fluid can lead to blooms of certain bacterial taxa, which have to be removed from the data prior to comparing microbial communities between samples. The ability to account for these blooms suggests that future microbiome studies may be able to use the dry-swab technique and obtain reliable data. This issue reflects the challenge of balancing the quality of samples with citizen science accessibility to an easy and inexpensive sample submission kit. Other challenges are common to large citizen science data sets. For example, the study is not specifically designed for the purpose of testing certain questions, thus reducing the sample size that can be used for specific analyses. These projects also usually have participant biases, with the majority of samples coming from Western populations and from individuals of higher education and income levels who live in cities. Nevertheless, the American Gut data set is large and diverse enough that these challenges do not limit the ability to detect novel biological patterns.

Even in its preliminary stages, the American Gut Project has expanded our knowledge of the structure and function of the gut microbiome. Further, this work illuminates the crucial role of citizen science in compiling large, multifaceted data sets. As more samples are added to the collection, it has great potential to further our understanding of the complex relationships between hosts, microbes, and health.
