# Erratum for Grieneisen and Blekhman, “Crowdsourcing Our National Gut”

**DOI:** 10.1128/mSystems.00114-18

**Published:** 2018-07-10

**Authors:** Laura E. Grieneisen, Ran Blekhman

**Affiliations:** a Department of Genetics, Cell Biology, and Development, University of Minnesota, Minneapolis, Minnesota, USA; b Department of Ecology, Evolution, and Behavior, University of Minnesota, Minneapolis, Minnesota, USA

## ERRATUM

Volume 3, no. 3, e00060-18, 2018, https://doi.org/10.1128/mSystems.00060-18. Page 1 of the PDF incorrectly stated the Published date and was corrected on 27 June 2018. The correct Published date is 15 May 2018, not 9 May 2018.

